# Developmental venous anomaly associated ischemic stroke caused by minor head trauma: A case report

**DOI:** 10.1097/MD.0000000000022305

**Published:** 2020-09-18

**Authors:** Chan-Hyuk Lee, Byoung-Soo Shin, Hyun Goo Kang

**Affiliations:** a Department of Neurology and Research Institute of Clinical Medicine of Jeonbuk National University, Deokjin-gu, Jeonju; b Biomedical Research Institute, Jeonbuk National University Medical School and Hospital, Deokjin-gu, Jeonju, Republic of Korea.

**Keywords:** developmental venous anomaly, head trauma, ischemic stroke

## Abstract

**Rationale:**

A developmental venous anomaly (DVA) is the most common intracranial congenital anomaly and is mostly asymptomatic. Thrombosis rarely develops in a DVA due to hypercoagulation. We report a case of ischemic stroke in the area of a DVA after minor head trauma in a patient with DVA and without a predisposition thrombosis.

**Patient concerns:**

A healthy 17-year-old male was admitted to the emergency room due to left hemiparesis, which was caused by a ball hitting the right side of his head during a soccer game.

**Diagnosis:**

Brain magnetic resonance (MR) susceptibility-weighted image showed several small veins draining to the central vein in the area from the right posterior putamen to the periventricular white matter.

**Interventions:**

We diagnosed the patient with an ischemic stroke associated with a DVA and administered antiplatelet agents. The patient's autoantibodies (including antiphospholipid antibody) and factors of blood coagulation were normal.

**Outcomes:**

The left hemiparesis of the patient worsened by the second day of admission. Moreover, high signal intensity was observed in the DVA region of the diffusion weighted image of brain MR. The patient's symptoms gradually improved afterward, and left hemiparesis recovered fully 3 weeks after the onset.

**Lessons:**

DVAs may predispose to ischemic stroke due to thrombosis and hypercoagulation, although it is rare. It is necessary to consider the possibility of ischemic stroke due to minor head trauma, even without factors causing hypercoagulation.

## 1 Introduction

According to a study conducted in the United States, there are 130.8 head injuries per 100,000 people per year and ∼80% of these cases are associated with mild injuries.^[1]^ Most patients were under 25 years old and ∼150 cases per 100,000 people per year occurred in children below 5 years old. A few studies^[2,3]^ have reported that teenagers who have experienced head trauma may suffer from ischemic stroke due to injury of cerebral artery.

A developmental venous anomaly (DVA) refers to abnormal development of the cerebral veins. A DVA is the most common congenital vascular malformation in the cranial cavity. Most DVA cases are asymptomatic, but rarely are associated with venous infarction caused by thrombosis due to a hypercoagulable state. There are case reports showing that patients with a DVA have experienced cavernous malformation (CM) rupture due to head trauma.^[4]^ However, no study has reported a vascular complication due to trauma in patients with an isolated DVA.

In this case, ischemic stroke occurred in the area of the DVA due to a minor head trauma without any underlying cause of thrombosis. We suggest that minor head trauma could be a causal factor for ischemic stroke associated with a DVA.

## 2 Case report

A healthy 17-year-old male was admitted to the emergency room due to left hemiparesis, which was caused by a ball hitting the right side of his head during a soccer game. The symptoms occurred suddenly 10 min after the impact, and he visited the nearest hospital before referral to our hospital. According to the neurological examination at the first hospital, hypesthesia was observed in the left face and arm of the patient. Weakness of his left upper and lower extremity (Medical Research Council scale, MRC grade 1) was also identified (National Institutes of Health Stroke Scale, NIHSS 9). At 1 h and 30 min after the onset of the neurological symptoms, no hemorrhage was found on brain computed tomography (CT). Therefore, intravenous tissue plasminogen activator (t-PA) was immediately administered and the patient was transferred to our hospital.

**Figure 1:**
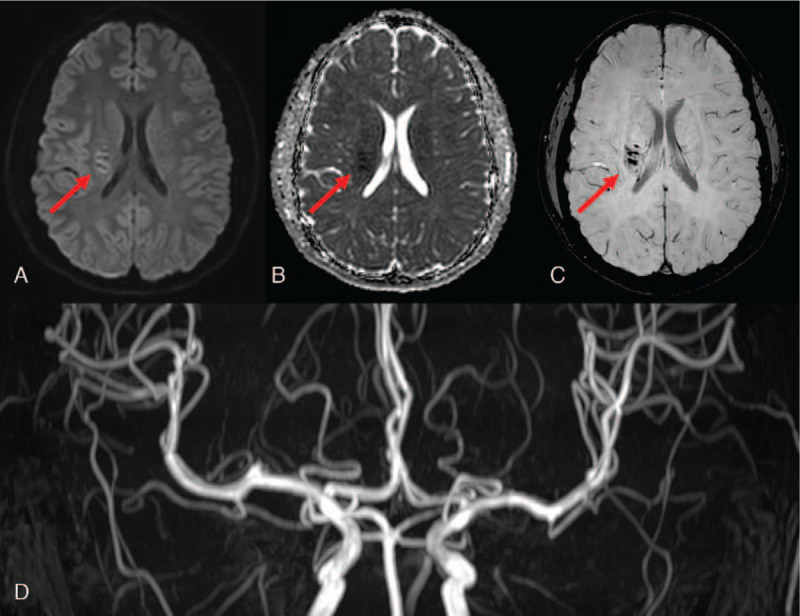
Brain magnetic resonance (MR) in a patient with left hemiparesis after minor head trauma. A developmental venous anomaly (DVA) with ischemic stroke was identified in the area of the right lenticulostriate artery. (All red arrows, A–C) (A) diffusion-weighted image; (B) apparent diffusion coefficient; (C) susceptibility-weighted image; and (D) normal findings of brain MR angiography.

When the patient was admitted, his vital signs were stable. Neurological examination revealed that the hypesthesia of his left face and arm was similar to the condition at the time of onset, but his left hemiparesis had improved to MRC grade 4 (NIHSS 3). A blood test showed that the D-dimer slightly increased to 1.05 mg/L, but the results of a complete blood count, liver function test, and electrolyte test were normal. Brain magnetic resonance (MR) susceptibility-weighted image (SWI) revealed many small veins draining to the central vein in the area from the right posterior putamen to the periventricular white matter (Fig. 1). Although not prominent, there was an increase in signal intensity around the DVA seen on diffusion weighted image (DWI) (Fig. 1). We diagnosed the patient with ischemic stroke associated with DVA and administered an antiplatelet drug from the next day.

**Figure 2:**
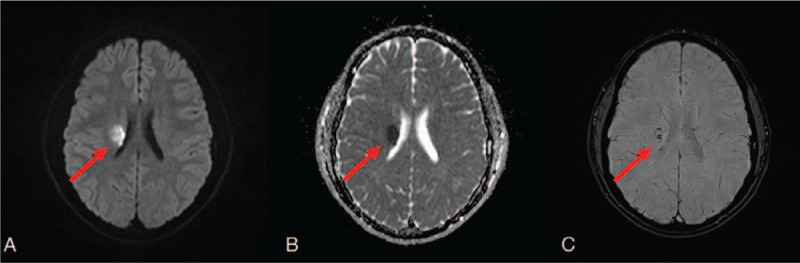
Brain MR 2 days after admission. In the diffusion-weighted image, the signal intensity of previous lesions is increased. (All red arrows indicate the acute ischemic lesions, A–C) (A) diffusion-weighted image; (B) apparent diffusion coefficient; and (C) susceptibility-weighted image.

The patient was further investigated for risk factors associated with young age-related stroke. There were normal levels of autoantibodies and coagulation factors including antiphospholipid antibodies. No abnormality was found in a transthoracic echocardiography. His left hemiparesis had worsened to MRC grade 3 from MRC grade 4 on the second day of hospitalization. Subsequent brain MR DWI showed that the high signal intensity became more prominent and broader in the area of the DVA (Fig. 2). Afterward, the patient's symptoms began to improve gradually, and the left hemiparesis completely resolved 3 weeks after the symptom onset.

## 3 Discussion

A DVA is the most common congenital vascular malformation that occurs in the cranial cavity, with an incidence rate of 0.5% to 2.7%.^[5]^ Most DVAs are asymptomatic, and are usually found by accident while undergoing a brain CT or MR scan during routine a medical checkup. Generally, an asymptomatic DVA is just observed without surgical intervention.

DVAs rarely cause clinical complications but symptomatic intracranial hemorrhage can be one of the major complications.^[5,6]^ This is often caused by accompanied cavernous malformation (CM). Meng et al^[7,8]^ reported that 11% of patients with a DVA had a CM as well. Unlike the DVA, which has the histological properties of normal veins, the CM has a higher a probability of rupture because it has abnormally expanded capillaries without an elastin layer. Additionally, thrombosis in the DVA can cause venous infarction with hemorrhage.^[9]^ Contraceptives^[10]^ and protein S deficiency^[9]^ have been reported as triggers for a hypercoagulable state associated with DVA. On the other hand, only a few reports (about 10) are available regarding ischemic stroke associated with DVA. Particularly, there was only one case which had no predisposition to thrombosis.^[11]^ The patient in this case was also a healthy 17-year-old male with no underlying disease or history of medication. Moreover, all results associated with coagulation factors were negative. There was just mild head trauma before neurological deficits.

Several cases have reported head trauma resulting in ischemic stroke. In these cases, ischemic stroke after blunt head trauma occurred mostly in infants 1 year or younger. It has frequently been observed in the basal ganglia (BG) or the internal capsule (IC). Balachandran et al reported that right hemiparesis developed after a 10-month-old girl fell and bumped her head.^[12]^ She was diagnosed with ischemic stroke in the left BG and IC. The researcher suggested that vascular damage due to the anatomical characteristics of the cerebral artery was a causative factor. Most trauma-related ischemic strokes occur in infants around 1-year-old, but Kargl et al reported a trauma-related ischemic stroke in a teenager.^[13]^ He reported an ischemic stroke in the right BG and parahippocampus of a 10-year-old girl and suggested a similar mechanism to the previous cases.

Several mechanisms have been suggested to explain how physical trauma causes an ischemic stroke. The most convincing hypothesis is that the ischemic stroke is caused by the anatomical characteristics of cerebral artery, as mentioned earlier. Blood is supplied to the BG and IC through the lenticulostriate artery from the middle cerebral artery. As this artery is a perforating artery, it branches off at acute angles in the parent artery. Therefore, if the brain parenchyma is suddenly shaken due to an external force, the blood supply to the perforating artery is likely to decrease temporarily.^[14]^ Meanwhile, it is suspected that the sudden movement of brain tissue can damage the intimal layer of blood vessels and it can trigger thrombosis.^[14]^ Particularly, people in the adolescent period or younger may be more vulnerable to the external force because their cerebral vessels are maturing. It is noteworthy that our patient had a venous anomaly from the right putamen to IC, unlike other case studies. DVAs, unlike arteries, do not have an elastic layer, making them more vulnerable to an external force. In other words, the shear stress caused by an impact may cause minor injury inside the vein and this may cause thrombosis. This is supported by the fact that the patient's neurological deficits occurred 10 min (a delayed onset) after the head trauma.

Although DVAs are usually asymptomatic, ischemic strokes due to thrombosis of a DVA can occur in a hypercoagulable state. Additionally, minor head trauma may induce DVA-associated ischemic stroke, even if there is no other cause for the hypercoagulable state.

## Author contributions

CHL, BSS, and HGK participated the design of this research. CHL and BSS collected and analyzed the raw clinical data. CHL and HGK carried out computational studies and wrote the manuscript. All authors have read and approved the final manuscript.

**Conceptualization:** Chan-Hyuk Lee, Hyun Goo Kang.

**Data curation:** Chan-Hyuk Lee, Hyun Goo Kang.

**Formal analysis:** Byoung-Soo Shin.

**Funding acquisition:** Hyun Goo Kang.

**Investigation:** Chan-Hyuk Lee.

**Methodology:** Byoung-Soo Shin.

**Supervision:** Byoung-Soo Shin, Hyun Goo Kang.

**Validation:** Byoung-Soo Shin.

**Visualization:** Chan-Hyuk Lee, Hyun Goo Kang.

**Writing – original draft:** Chan-Hyuk Lee, Hyun Goo Kang.

## Abbreviations

Abbreviations: BG = basal ganglia, CM = cavernous malformation, CT = computed tomography, DVA = developmental venous anomaly, DWI = diffusion weighted image, IC = internal capsule, MR = magnetic resonance, MRC = Medical Research Council scale, NIHSS = National Institutes of Health Stroke Scale, SWI = susceptibility-weighted image, t-PA = tissue plasminogen activator, VBI = vertebrobasilar insufficiency.
